# Maternal acrylamide exposure changes intestinal epithelium, immunolocalization of leptin and ghrelin and their receptors, and gut barrier in weaned offspring

**DOI:** 10.1038/s41598-023-37590-3

**Published:** 2023-06-24

**Authors:** Siemowit Muszyński, Monika Hułas-Stasiak, Piotr Dobrowolski, Marcin B. Arciszewski, Ligia Hiżewska, Janine Donaldson, Sylwia Mozel, Karol Rycerz, Małgorzata Kapica, Iwona Puzio, Ewa Tomaszewska

**Affiliations:** 1 Department of Biophysics, Faculty of Environmental Biology, University of Life Sciences in Lublin, Akademicka St. 13, 20-950 Lublin, Poland; 2 Department of Functional Anatomy and Cytobiology, Faculty of Biology and Biotechnology, Maria Curie-Sklodowska University, Akademicka St. 19, 20-033 Lublin, Poland; 3 Department of Animal Anatomy and Histology, Faculty of Veterinary Medicine, University of Life Sciences in Lublin, Akademicka St. 12, 20-950 Lublin, Poland; 4 School of Physiology, Faculty of Health Sciences, University of the Witwatersrand, 7 York Road, Parktown, Johannesburg, 2193 South Africa; 5 Department of Animal Physiology, Faculty of Veterinary Medicine, University of Life Sciences in Lublin, Akademicka St. 12, 20-950 Lublin, Poland

**Keywords:** Nutrition, Public health

## Abstract

Acrylamide (ACR) is an amide formed as a byproduct in many heat-processed starchy-rich foods. In utero ACR exposure has been associated with restricted fetal growth, but its effects of postnatal functional development of small intestine is completely unknown. The current study investigated the time- and segment-dependent effects of prenatal ACR exposure on morphological and functional development of small intestine in weaned rat offspring. Four groups of pregnant female Wistar rats were exposed to ACR (3 mg/kg b.w./day) for 0, 5, 10 and 15 days during pregnancy. Basal intestinal morphology, immunolocalization of gut hormones responsible for food intake and proteins of intestinal barrier, activity of the intestinal brush border disaccharidases, apoptosis and proliferation in intestinal mucosa were analyzed in offspring at weaning (postnatal day 21). The results showed that in utero ACR exposure disturbs offspring gut structural and functional postnatal development in a time- and segment-depended manner and even a short prenatal exposure to ACR resulted in changes in intestinal morphology, immunolocalization of leptin and ghrelin and their receptors, barrier function, activity of gut enzymes and upregulation of apoptosis and proliferation. In conclusion, prenatal ACR exposure disturbed the proper postnatal development of small intestine.

## Introduction

Acrylamide (ACR; prop-2-enamide or acrylic amide) is found in various paints, plastics, varnishes, mortar, adhesives, toiletries and cosmetics^1^. ACR is naturally formed through the interaction of amino acids with reducing sugars like asparagine and glucose during frying and baking^2^. ACR is also detected in thermally processed animal feed and ACR carry-over from livestock animals to eggs or milk has been reported^3–6^.

International Agency for Research on Cancer (IARC) and UE^7^ regard ACR as a chemical hazard in food and consider it a reproductive and mutagenic toxin, making it a group 2A carcinogen. In animals, ACR at neurotoxic doses causes a reduction in food and water intake, leading to decrease in body weight and lactation rate^8,9^.

Prenatal nutrition is important in the healthy physical and mental development of mammals and has long-term effects evident later in life^10,11^. ACR is often consumed unknowingly and thus ACR dietary intake is difficult to assess^12–14^. Infants, toddlers and other children are amongst the most ACR-exposed^12^. Upon dietary intake, ACR is absorbed from the gastrointestinal tract and distributed into the tissues, transferred into milk and crosses the placenta^12^ reaching the fetuses^9^. Maternal nutrition affects fetal development, metabolism, physiology, and genome expression^10,15–19^.

Enteroplasticity following prenatal ACR exposure has been extensively studied, however, much less is known regarding the time-dependent effects of prenatal ACR exposure. To our knowledge, there are no studies that have investigated ACR effects on prenatal small intestine (SI) development (immunolocalization of gut hormones, as well as tight and adherent type cell–cell junctions).

**Figure 1:**
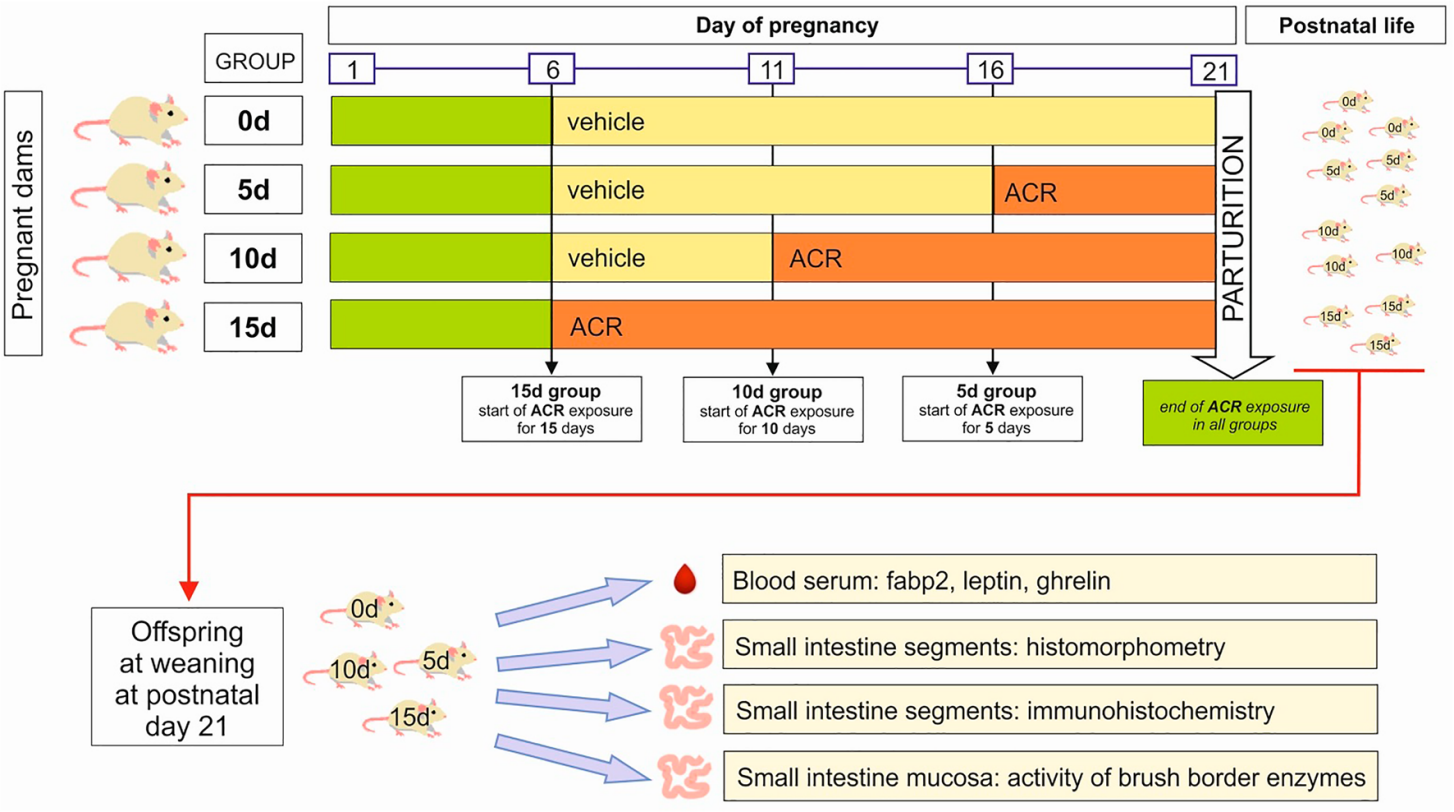
(**A**) Scheme of the experimental design. Pregnant dams were not exposed (the 0d group) or exposed to ACR (3 mg/kg b.w./day, administrated by oral gavage) for a period of either 15 days, (the 15d group), 10 days (the 10d group) or 5 days (the 5d group), from the 6th, 11th or 16th day of pregnancy until parturition, respectively. On postnatal day 21 (weaning), n = 12 offspring from each experimental group were sacrificed and blood and small intestine samples were collected for analyses.

The aim of the current study was to determine the effects of maternal ACR exposure (Fig. 1) on offspring`s SI development at weaning. To provide an adequate explanation for what occurs after prenatal ACR exposure, the study examined the time- and segment-dependent effects of ACR on intestine morphology; immunolocalization of ghrelin and leptin, and proteins forming adherent and tight junctions (E-cadherin, occludin) in a rat model.

## Results

Table 1 shows statistically significant changes in morphology of SI sections. The results of whole histomorphometry analysis are presented in Supplementary Table S1.

**Table 1:** Statistically significant changes in basal morphological parameters of small intestine sections of weaned Wistar rats not prenatally exposed to acrylamide (0d) or exposed to acrylamide for 5, 10 or 15 days of prenatal life up to parturition.

	0d	5d	10d	15d	*p* value
Duodenum					
Submucosa thickness, μm	22.62 ± 2.52^a^	24.08 ± 1.51^ab^	38.96 ± 6.86^b^	18.06 ± 1.26^a^	0.005
Villus length, µm	647 ± 4^c^	479 ± 4^a^	555 ± 7^b^	673 ± 12^c^	< 0.001
Villus thickness, µm	78.68 ± 1.67^c^	35.20 ± 0.97^a^	69.75 ± 1.96^b^	79.66 ± 1.08^c^	< 0.001
Villus volume, µm^3^	159.9 ± 3.3^c^	53.0 ± 1.5^a^	121.4 ± 3.2^b^	168.5 ± 2.5^c^	< 0.001
Enterocyte number, (100 µm of villus)^-1^	18.72 ± 0.33^ab^	17.12 ± 0.43^a^	19.06 ± 0.57^b^	18.56 ± 0.52^ab^	0.042
Crypt depth, μm	134.7 ± 1.0^c^	56.26 ± 3.56^a^	88.96 ± 1.62^b^	140.99 ± 2.48^c^	< 0.001
Crypt thickness, µm	45.27 ± 0.72^d^	26.60 ± 0.54^b^	20.69 ± 0.21^a^	39.76 ± 0.30^c^	< 0.001
Villus:crypt ratio, –	4.81 ± 0.04^a^	9.05 ± 0.72 ^b^	6.26 ± 0.16 ^a^	4.80 ± 0.16 ^a^	< 0.001
Mucosal surface absorptive area, –	13.92 ± 0.17^a^	18.58 ± 0.25^c^	19.59 ± 0.51^c^	15.72 ± 0.27^b^	< 0.001
Proximal jejunum					
Total number of villi, mm^-1^	7.92 ± 0.53^a^	9.75 ± 0.66^ab^	10.0 ± 0.69^ab^	10.83 ± 0.52^b^	0.040
Enterocyte number, (100 µm of villus)^-1^	16.13 ± 0.37^a^	17.89 ± 0.40^b^	18.74 ± 0.53^b^	18.55 ± 0.38^b^	0.001
Total crypts number, mm^-1^	2.64 ± 0.15^a^	3.28 ± 0.13^b^	3.29 ± 0.13^b^	2.95 ± 0.16^ab^	0.021
Inactive crypts number, mm^-1^	2.30 ± 0.18^a^	3.00 ± 0.11^b^	3.01 ± 0.14^b^	2.67 ± 0.13^ab^	0.008
Crypt depth, μm	111.7 ± 6.4^c^	94.4 ± 4.4^bc^	86.8 ± 4.3^ab^	69.1 ± 3.6^a^	< 0.001
Crypt thickness, µm	34.80 ± 1.18^b^	25.99 ± 0.93^a^	26.36 ± 0.60^a^	28.34 ± 1.24^a^	< 0.001
Villus:crypt ratio, –	3.33 ± 0.24^a^	4.15 ± 0.26^ab^	4.51 ± 0.20^ab^	5.51 ± 0.62^b^	0.005
Middle jejunum					
Submucosa thickness, μm	15.77 ± 0.93^b^	9.39 ± 0.59^a^	13.37 ± 1.33^ab^	15.03 ± 1.35^b^	0.049
Villus length, µm	345.8 ± 17^ab^	286.4 ± 12^a^	315.5 ± 10^ab^	362.5 ± 25^b^	0.025
Enterocyte number, (100 µm of villus)^-1^	18.00 ± 0.32^a^	19.93 ± 0.40^b^	19.19 ± 0.40^a^	18.58 ± 0.24^a^	0.006
Crypt depth, μm	113.94 ± 6.84^b^	88.75 ± 7.47^a^	86.28 ± 4.51^a^	95.25 ± 3.39^ab^	0.013
Crypt thickness, µm	35.35 ± 1.3^b^	27.67 ± 1.24^a^	27.71 ± 1.02^a^	27.69 ± 0.47^a^	< 0.001
Mucosal surface absorptive area, –	8.14 ± 0.40^ab^	7.97 ± 0.37^a^	8.73 ± 0.29^ab^	10.11 ± 0.98^b^	0.033
Distal jejunum					
Mucosa thickness, μm	310.7 ± 17^b^	308.7 ± 9^ab^	253.8 ± 9^a^	302.5 ± 11^ab^	0.031
Longitudinal lamina thickness, μm	15.84 ± 0.93^ab^	18.01 ± 1.34^b^	12.99 ± 0.67^a^	14.07 ± 0.58^ab^	0.016
Enterocyte number, (100 µm of villus)^-1^	18.51 ± 0.62^a^	19.91 ± 0.34^a^	21.89 ± 0.35^b^	20.22 ± 0.23^a^	< 0.001
Total crypts number, mm^-1^	2.58 ± 0.11^a^	3.05 ± 0.14^b^	2.82 ± 0.12^a^	2.99 ± 0.06^a^	0.036
Crypt depth, μm	110.96 ± 4. 94^c^	99.80 ± 2.20^bc^	82.41 ± 2.23^a^	89.34 ± 3.83^ab^	< 0.001
Villus:crypt ratio, –	2.12 ± 0.10^a^	2.36 ± 0.16^ab^	2.55 ± 0.11^ab^	2.72 ± 0.16^b^	0.038
Ileum					
Total number of villi, mm^-1^	4.92 ± 0.40^a^	7.25 ± 0.33^b^	6.50 ± 0.48^ab^	6.42 ± 0.48^ab^	0.008
Crypt thickness, µm	38.28 ± 1.22^b^	30.60 ± 1.38^a^	33.14 ± 1.79^ab^	31.68 ± 1.43^a^	0.015

Data are presented as lsmeans ± SEM (standard error of mean). Rows with different superscripts (^a, b, c^) are significantly different at *p*-value < 0.05 (GLM MIXED procedure with an individual rat as the experimental unit, n = 12 per group, and a post hoc Tukey’s HSD adjustment).

### Intestinal basal morphology

#### Duodenum

The 10d and 15d ACR exposure had the most significant effect on duodenal morphology, reducing villus length, thickness and volume and crypt depth and thickness (Table 1). Increased villus height/crypt depth ratio (VH:CD ratio) was observed in the 5d group (shortest ACR exposure). Additionally, a reduction in crypt thickness and an increase in mucosal surface absorptive area, expressed as mucosal-to-serosal amplification ratio, was noted in all ACR exposed groups, with the most significant increase observed in the 10d and 15d groups.

#### The proximal jejunum (PJ)

ACR exposure increased the number of enterocytes in the villi of all groups exposed to ACR and the number of villi in the 15d group, when compared to the control (Table 1). An increase in number of total and inactive crypts was observed in the 10d and 15d groups and the crypt thickness was reduced in all ACR-exposed groups compared to the control. Crypt depth in the 10d and 15d groups was also lower compared to the control, and for the 15d group also when compared to the 5d group. The VH:CD ratio was significantly higher in the 15d group compared to 0d group.

#### The middle jejunum (MJ)

Submucosa thickness was lower in the 5d group compared to the 0d and 15d groups (Table 1). In turn, while villi length in the 5d group was lower only when compared to the 15d group, the number of enterocytes per 100 µm of villus noted in that group was higher compared to all other groups. ACR exposure decreased crypt thickness in all ACR-exposed groups compared to the control, but a decrease in crypt depth was only observed in the 5d and 10d groups.

#### The distal jejunum (DJ)

Mucosa thickness was lower in the 10d group compared to the control, while longitudinal lamina thickness was lower compared to the 5d group (Table 1). ACR exposure increased villi enterocyte number of the 10d group. Increased number of crypts was noted in the 5d group, compared to all other groups. A time-dependent effect of prenatal ACR-exposure was observed in crypt depth, with lower crypt depth observed in the 10d and 15d groups compared to the 0d group. However, a change in VH:CD ratio was only observed in the 15D group, compared to the control.

#### Ileum

Villi number was higher in the 5d group compared to the 0d group and villus epithelium thickness was higher in the 5d group compared to the 10d group (Table 1). Crypt thickness was lower in the 5d and 15d groups than in the 0d group (Table 1).

### Proliferating cells, TUNEL analysis and collagen distribution

**Figure 2:**
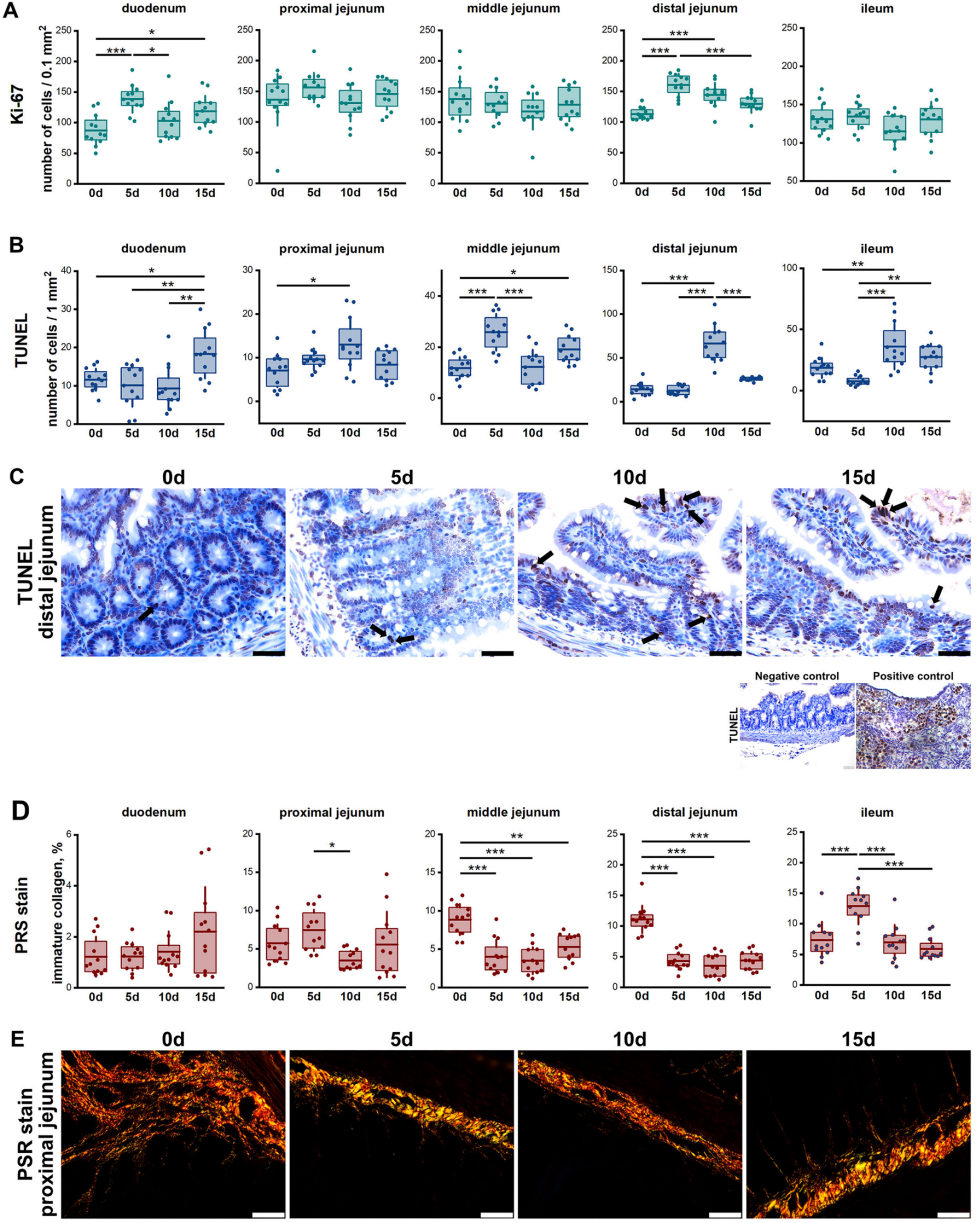
(**A**) Quantitative analysis of number of cells with positive Ki-67 staining per area of the gland surface. (**B**) Quantitative analysis of number of apoptotic cell by TUNEL assay. (**C**) Representative images of TUNEL reaction in distal jejunum. Apoptotic cells are marked as dark brown after TUNEL staining as observed under a light microscope. Arrows indicate representative TUNEL-positive cells in TUNEL reaction. All scale bars represent 40 µm. Positive control: Apoptotic oocytes are visible within egg nests of the piglet’s ovary. (**D**) Content of immature collagen fibers in small intestine sections as quantified by analysis of PRS stained sections. (**E**) Representative images of the distribution of immature collagen fibers (seen as green) in PRS-stained sections of proximal jejunum observed in polarized light. (**C**), (**E**): All scale bars represent 40 µm. (**A**), (**B**), (**D**): Box-plots show lsmeans value (line), interquartile range (box) and standard deviation (whiskers). A *p* value range was attributed above plots when two groups exhibit significant differences: *p value < 0.05, **p value < 0.01, ***p value < 0.001 (GLM MIXED procedure with an individual rat as the experimental unit, n = 12 per group, and a post hoc Tukey’s HSD adjustment).

Prenatal ACR exposure effects on number of proliferating cells was limited to the duodenum (Fig. 2A). Proliferating cells were increased in the 5d group compared to the 0d and 10d groups and in the 15d group compared to the 0d group. In the DJ, an increase was observed in the 5d and 10d groups, compared to the control; the 5d group also had an increased number of proliferating cells compared to the 15d group.

With regards to the apoptotic cells (AC) in the duodenum, only the 15d ACR-exposure group showed an increase (Fig. 2B). In the PJ, the number of AC in the unexposed group was lower, only when compared to the 10d group. In turn, in the MJ, the number of AC in the 0d and 10d groups was not different, but an increase in AC was observed in the 5d and 15d groups, compared to the control. In the DJ, an increase in AC number was observed in the 10d group only (Fig. 2C). In the ileum, AC number was increased in the 10d group compared to the 0d and 5d groups, and the AC number noted in the 5d group was also lower than that of the 15d group (Fig. 2B, C).

No differences in the content of immature collagen (IC) in the duodenum was observed between groups (Fig. 2D). In the PJ, a decreased IC content was observed in the 10d group compared to the 5d group (Fig. 2E). A decreased IC content was observed in the MJ and DJ in all prenatally ACR-exposed groups, compared to the control (Fig. 2D). In the ileum, a higher IC content was observed in the 5d group compared to all other groups.

### Intensity of immunoreaction

#### Ghrelin

**Figure 3:**
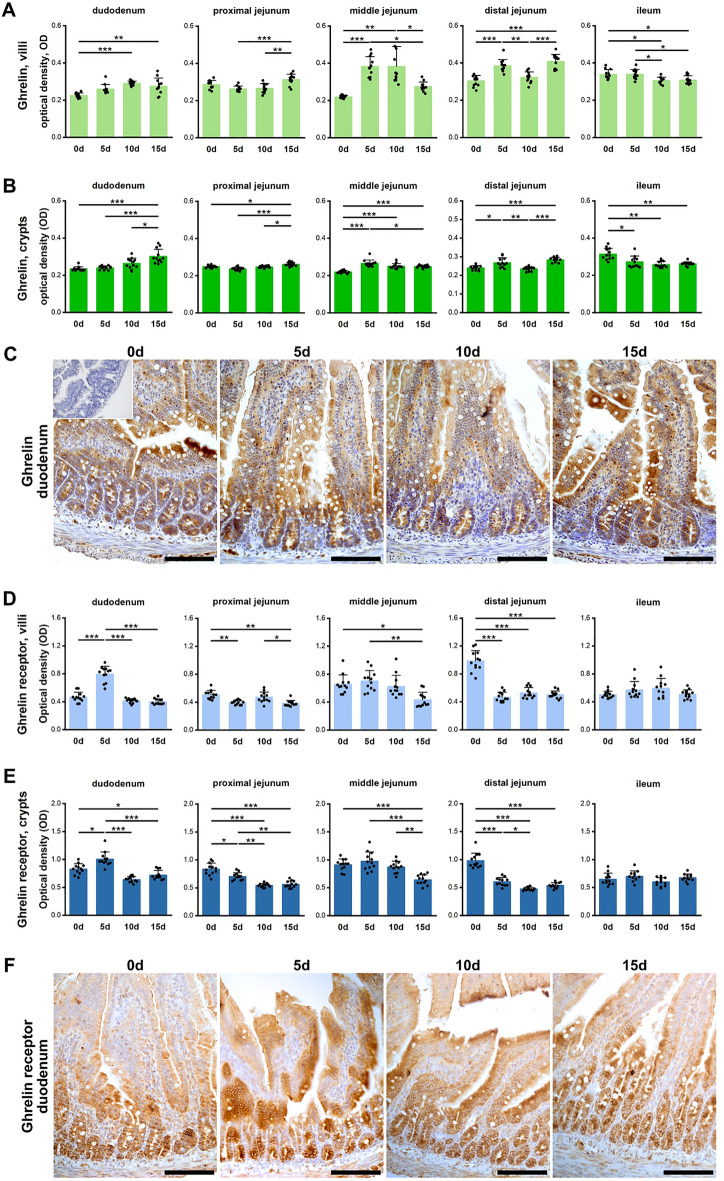
Quantitative analysis of the intensity of ghrelin IR in (**A**) villi and (**B**) crypts of small intestine segments, measured by comparison of the pixel brightness value in the microscopic images converted to 8-bit, grey-scale images (the higher the pixel value, the higher the intensity of the IR). (**C**) Representative images of the ghrelin IR in sections of proximal jejunum; inset: antibody control. Quantitative analysis of the intensity of ghrelin receptor IR in (**D**) villi and (**E**) crypts of small intestine segments, measured by comparison of the optical density (OD) of the microscopic images converted to 8-bit, grey-scale images. (**F**) Representative images of the ghrelin IR in sections of duodenum. (**C**), (**F**): All scale bars represent 100 µm. (**A**), (**B**), (**D**), (**E**): Bar plots show lsmeans value and standard deviation (whiskers). A *p*-value range was attributed above plots when two groups exhibit significant differences: *p value < 0.05, **p value < 0.01, ***p value < 0.001 (GLM MIXED procedure with an individual rat as the experimental unit, n = 12 per group, and a post hoc Tukey’s HSD adjustment).

The intensity of the ghrelin immunoreaction (IR) in the duodenal villi was stronger in the 10d and 15d groups (Fig. 3A), while in the crypts the ghrelin IR was significantly stronger in the 15d group compared to all other groups (Fig. 3B, C). The ghrelin IR in the villi of the PJ in the 15d group was stronger compared to that of the 5d and 10d groups, while in crypts it was the strongest. In the MJ villi of the 0d and 5d groups, the ghrelin IR was significantly weaker than that in the other two groups. In the crypts, it was significantly weaker in the 0d group compared to all prenatally ACR-exposed groups, with the difference noted between the 5d and 15d groups. The general pattern of ghrelin IR in the DJ was similar in the villi and crypts, with a weaker IR noted in the 0d and 10d groups. In turn, a stronger IR was observed in ileum villi of the 0d and 5d groups than in the 10d and 15d groups, while the ghrelin IR was weaker in the crypts of all parentally ACR-exposed groups compared to the control.

#### Ghrelin receptor

The strongest ghrelin receptor IR intensity was observed in the duodenal villi and crypts of the 5d group (Fig. 3D, E, F). In the crypts, the IR in the 0d group was also higher compared to that of 15d group. In the villi of the PJ, the IR in the 0d group was stronger than in the 5d and 15d groups, and that in the 10d group was stronger compared to that in the 15d group. In the PJ crypts, the strongest IR was observed in the 0d group, followed by the 5d group. In the villi and crypts of the MJ, a weaker intensity ghrelin receptor IR was observed in the 15d group compared to the 0d and 5d groups. The strongest ghrelin receptor IR in the DJ was observed in the control group. No changes were observed neither in the villi or crypts of the ileum in all groups.

#### Leptin

**Figure 4:**
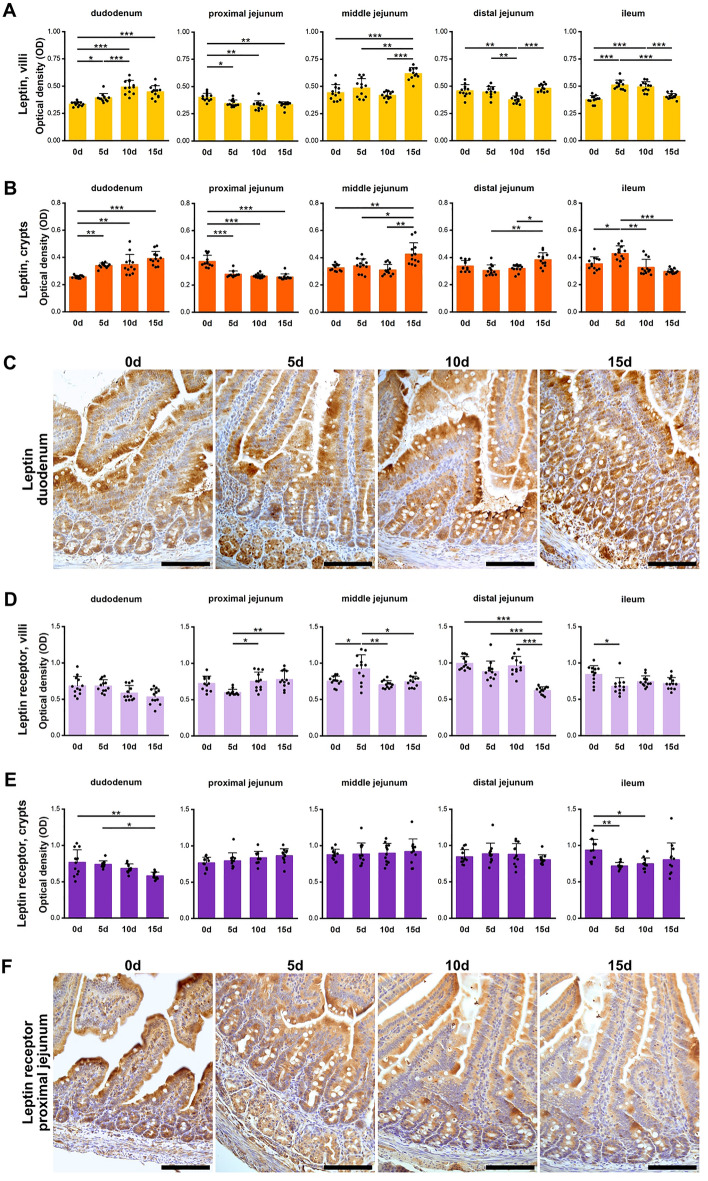
Quantitative analysis of the intensity of leptin IR in (**A**) villi and (**B**) crypts of small intestine segments, measured by comparison of the optical density (OD) of the microscopic images converted to 8-bit, grey-scale images. (**C**) Representative images of the leptin IR in sections of duodenum. Quantitative analysis of the intensity of leptin receptor IR in (**D**) villi and (**E**) crypts of small intestine segments, measured by comparison of the optical density (OD) of the microscopic images converted to 8-bit, grey-scale images. (**F**) Representative images of the ghrelin IR in sections of proximal jejunum. (**C**), (**F**): All scale bars represent 100 µm. (**A**), (**B**), (**D**), (**E**): Bar plots show lsmeans value and standard deviation (whiskers). A *p*-value range was attributed above plots when two groups exhibit significant differences: *p value < 0.05, **p value < 0.01, ***p value < 0.001 (GLM MIXED procedure with an individual rat as the experimental unit, n = 12 per group, and a post hoc Tukey’s HSD adjustment).

The weakest leptin IR intensity was observed in the duodenal villi and crypts of the control group (Fig. 4A–C). In the PJ, the leptin IR in the 0d group was significantly stronger than that in all prenatally ACR-exposed groups, while in the MJ, a significantly stronger leptin IR was observed in the 15d group compared to all other groups. In the villi of the DJ, a weaker leptin IR was observed in 10d group, while in crypts the IR in the 15d group was stronger than that in the 5d and 10d groups. In the ileum, a stronger leptin IR was observed in both the 5d and 10d group (villi) and in the 5d group (crypts), compared to all other groups.

#### Leptin receptor

There was no effect of ACR-exposure on leptin receptor IR in duodenal villi (Fig. 4D). In the crypts of the duodenum, the IR for the leptin receptor was lower in the 15d group that of the 0d and 5d groups (Fig. 4D, E). In the crypts of all jejunal segments, no differences in IR intensity were observed. In the villi of the PJ, the IR for the leptin receptor in the 5d group was weaker compared to that of the 10d and 15d groups (Fig. 4F), while in the villi of MJ, the IR in the 5d group was stronger than in the other groups. In the villi of the DJ, a significantly weaker leptin IR was observed in the 15d group compared to all other groups. In the villi of the ileum, the leptin IR in the 0d group was stronger compared to the 5d group, while in the crypts it was stronger when compared to the 5d and 10d groups.

#### Occludin

**Figure 5:**
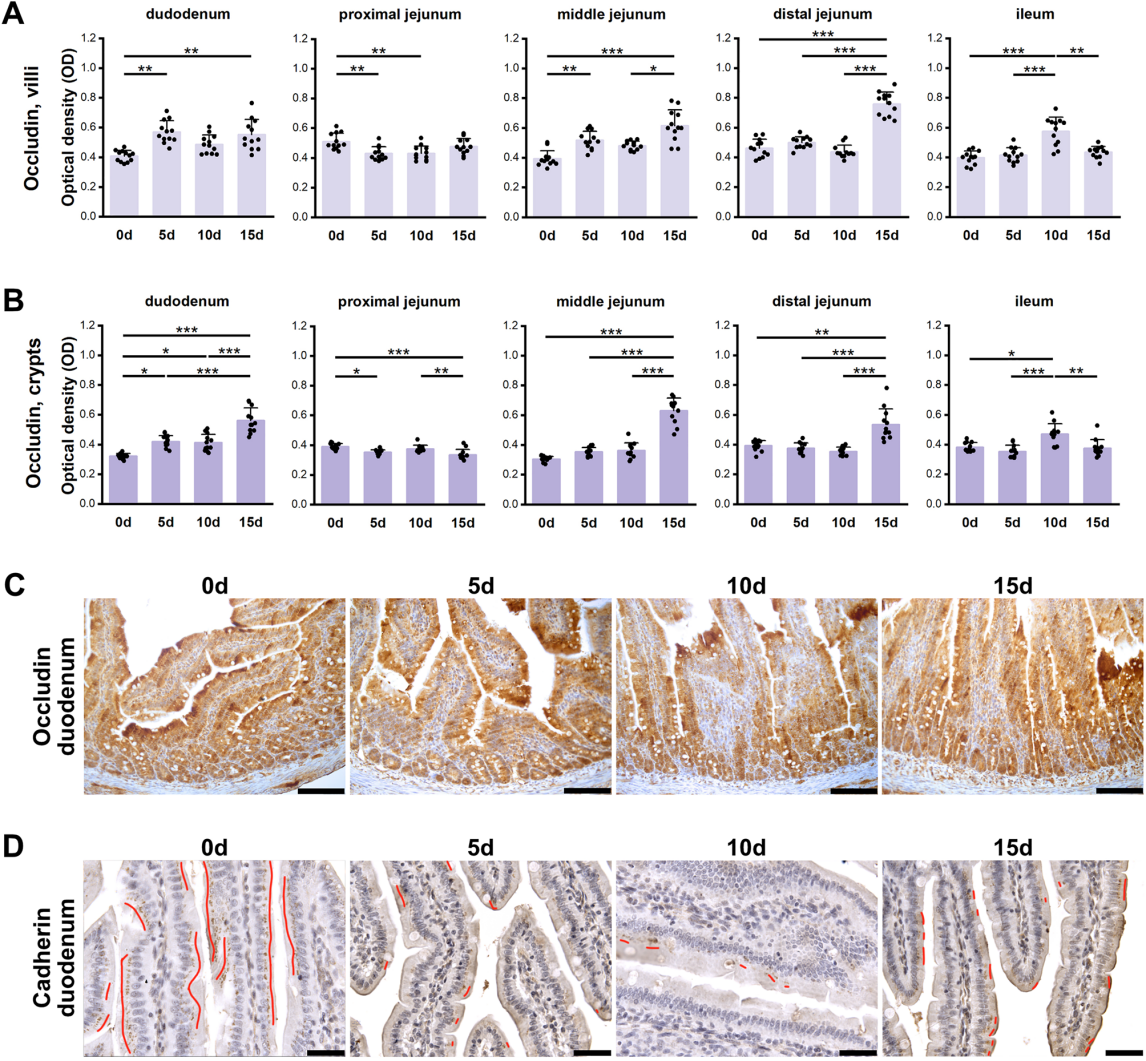
Quantitative analysis of the intensity of occludin IR in (**A**) villi and (**B**) crypts of small intestine segments, measured by comparison of the optical density (OD) of the microscopic images converted to 8-bit, grey-scale images. (**C**) Representative images of the occludin IR in sections of duodenum. All scale bars represent 100 µm. (**D**) Representative images of the cadherin IR in sections of duodenum. Red lines show the presence of cadherins. All scale bars represent 40 µm. A, B: Bar plots show lsmeans value and standard deviation (whiskers). A *p*-value range was attributed above plots when two groups exhibit significant differences: * *p* value < 0.05, ** *p* value < 0.01, *** *p* value < 0.001 (GLM MIXED procedure with an individual rat as the experimental unit, n = 12 per group, and a post hoc Tukey’s HSD adjustment).

The effect of prenatal ARC-exposure on the IR for occludin, a tight junction protein, was segment-dependent (Fig. 5A, B). In the duodenal villi, the occludin IR in the 0d group was weaker compared to that in the 5d and 15d groups. In the crypts, the IR in the control group was significantly weaker than in all prenatally ACR-exposed groups, while it was strongest in the 15d group (Fig. 5C). In the PJ villi, occludin IR in the 0d group was stronger than in the 5d and 10d groups, while in the crypts it was stronger when compared to the 5d and 15d groups. The occludin IR in the PJ crypts of the 15d group was also weaker than that in the 10d group. In the MJ villi, the weakest IR was observed in the 0d group, followed by the 10d group. In the MJ crypts, the 15d group had a stronger occludin IR than all other groups. The same was observed in both sections of the DJ, i.e. significantly stronger occludin IR in the 15d group. In the ileal villi and crypts, a stronger IR for occludin was observed in the 10d group compared to all other groups.

#### Cadherin

The IR intensity for cadherin, another tight junction protein, was continuous and well visible in the 0d group in all SI segments, while the IR was fragmented in all prenatally ACR-exposed rats, irrespective of length of exposure and intestine segment (Fig. 5D).

### The activity of mucosal brush border enzymes

**Figure 6:**
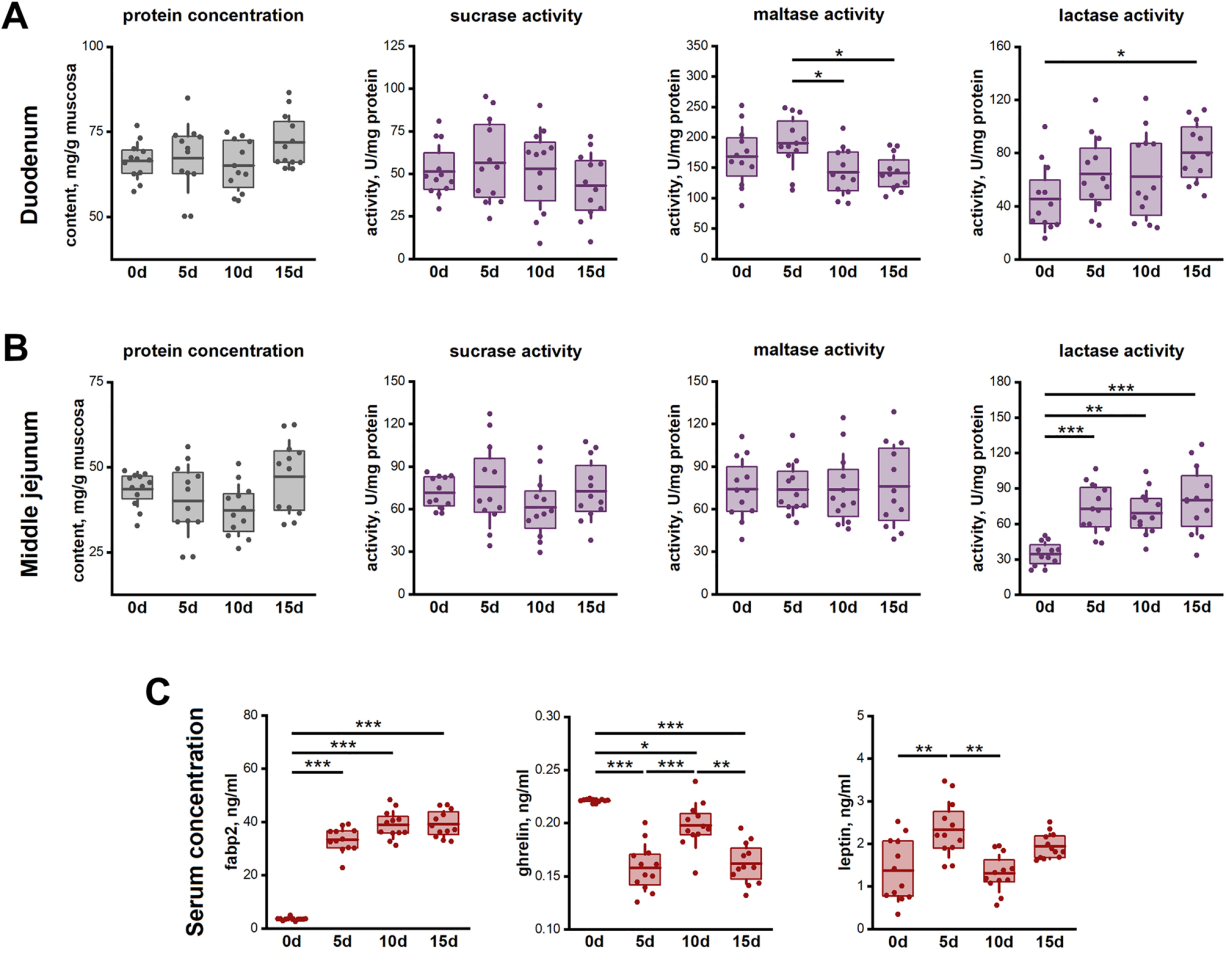
Protein concentration and enzyme activity of the intestinal brush border disaccharidases (sucrase, maltase, and lactase) in duodenum (**A**) and middle jejunum (**B**). (**C**) Blood serum concentrations of fatty acid binding protein 2 (fabp2), ghrelin, and leptin. Box-plots show lsmeans value (line), interquartile range (box) and standard deviation (whiskers). A *p* value range was attributed above plots when two groups exhibit significant differences: *p value < 0.05, **p value < 0.01, ***p value < 0.001 (GLM MIXED procedure with an individual rat as the experimental unit, n = 12 per group, and a post hoc Tukey’s HSD adjustment).

Prenatal ACR-exposure had no effect on protein concentration or sucrase activity in the duodenum and MJ (Fig. 6A). The maltase activity in the MJ was the same in all groups, while in the duodenum it was increased in the 5d group compared to all other ACR-exposed groups. Duodenal lactase activity in the control group was lower compared to the 15d group only, while in the MJ it was lower compared to all prenatally ACR-exposed groups (Fig. 6B).

### Serum analyses

The serum concentration of intestinal fatty acid-binding protein 2 (fabp2) was significantly increased in all prenatally ACR-exposed groups (Fig. 6C). The serum ghrelin concentration was highest in the control group, followed by the 10d group. The serum concentration of leptin was higher in the 5d group, compared to the 0d and 10d groups.

## Discussion

Prenatal development of the rat SI has been previously described in detail^20^. SI smooth muscle and epithelial cells are not yet differentiated on day 15.5, however the intestinal lumen is surrounded by cells forming tight junctions. Circular and longitudinal muscular layers develop on day 16.5. On day 17.5 the first duodenal villi appear, epithelial cells differentiate to enterocytes and the first endocrine cells develop. On day 18.5 the villi epithelium is complete with goblet cells. The last 3 days of prenatal development are crucial for complete intestine development, with intestinal mucosa which is comparable to that found postnatally, due to a significant increase in villi and microvilli number. Villi shape and size are also determined on day 21^20^. Newborn rats have a short intestine (20 cm), weighing about 0.066 g, and during the first 21 days of postnatal life there is a large increase in intestinal weight (about eightfold) and a twofold increase in length^21^.

The current study was designed to take place when the most important alterations occur in intestine development (the last 5 days) and showed that maternal ACR exposure reduced mucosa thickness in the DJ. A reduction in mucosa thickness could not only influence secretion, digestion and absorption, but also possibly disturb the protective functions of the intestine, involving non-specific (the defense against injury) and specific immunity (the content of mucosa-associated lymphoid tissue)^22^. The negative effects of ACR exposure are still not fully understood, specifically with regards to its developmental effects.

The only available study on prenatally ACR-exposed guinea pigs (3 mg/kg b.w./day) presents a similar reduction in jejunal mucosa thickness^19^. It should be emphasized that maternal ACR exposure was longer in the guinea pigs than that in the current rat-based study, due to the guinea pig pregnancy lasting between 68–70 days. In the current study the longest exposure lasted for 15 days. Another previous study observed a decrease in stomach mucosa thickness and the loss of normal architecture^23^, however the rats received 30 mg/kg of ACR for 4 weeks postnatally.

The spiral arrangement of smooth muscle fibers of the muscular lamina (ML), which consists of two layers (inner circular and outer longitudinal), determines the spiral passage of chyme to the periphery. The ML is thickest proximally and thins as it moves away from the pylorus. Taking into account the total thickness of the ML, this basal pattern was maintained in weaned, control rats in the current study, while time-dependent changes in the longitudinal layer of the DJ were observed.

Two types of movements are observed in GIT: propulsive and mixing movements. Mixing movements serve to mix the chyme with digestive juices and are found more in the PJ, while propulsive movements move chyme both forwards and backwards.

Thinning of the ML in prenatally ACR-exposed rats could disturb proper chyme movement, leading incomplete emptying and retention of content. Since the intestinal contraction wave, which propels chyme forwards, is dependent on longitudinal muscular layer function, the thinning of this layer in the DJ of rats after 10 days of prenatal ACR exposure, could compound these negative effects. Moreover, the basic electrical rhythm (BER) is triggered in the interstitial pacemaker cells of Cajal^24^. Although there is a gradient in BER frequency along the intestine, with the electrical activity in the ileum being the lowest, it is not known what changes could arise due to this thinning, since the physiological properties of the SI, such as excitability and contractility, are dependent on physical factors such as compression and stretching of the intestinal wall by the chyme, in which the muscular layer plays a significant role. For this reason, further studies are needed to clarify all the effects of its altered thickness.

Villi and crypts are covered by a mucus membrane consisting of a single layer of cells, which play a crucial role in secretion, digestion and absorption^25^. Maternal ACR exposure led to an increase in villi epithelium thickness and enterocyte number in the duodenum and jejunum, showing a time-dependent effect. Furthermore, maternal ACR exposure resulted in changes in mucosal brush border enzyme activity. The typical developmental pattern of the disaccharidases activity (maltase and sucrase) shows low activity during the first two weeks of neonatal life, followed by a significant increase in the subsequent next two weeks. Newborns do not exhibit detectable levels of disaccharidases during the first and second week of life, but reach adult levels by the end of the fourth week of postnatal time. This pattern differs from that of lactase and holds physiological significance. During suckling, lactase serves as the primary enzyme for carbohydrate digestion, while maltose, isomaltose and sucrose become important disaccharides after weaning^26^. In the present study, this expected normal developmental pattern should be already observed in all offspring since the presented weaned rats were 21 days old, whereas it is clearly visible in control offspring. However, lactase activity exhibited changes dependent on time and intestine segment. As lactase degrades lactose into glucose and galactose, it altered activity may contribute to the observed increase in glucose levels in the 10d ACR-exposed rats, as well as the decreased insulin concentration^27^. These effects could be attributed to ACR influence on pancreatic function and a reduction in beta cells^28^. ACR exposure resulted in increased maltase activity in the 5d group, while it showed a non-statistically significant decrease in maltase activity in the 15d group. Prolonged maternal ACR exposure may have led to a statistically significant decrease. Furthermore, it is unknown whether maltase activity decreased in other organs, such as a testis, where it plays a crucial role in testis development^29,30^. It is important to note that ACR is a widely distributed hazardous compound with reproductive toxicity, causing disruptions in numerous metabolic pathways in both human and animals^31^. Considering the normal developmental pattern of enzyme activity, it is noteworthy that ACR maintained high lactase activity compared to sucrase or maltase, which contrast with the expected developmental pattern. While it can be assumed that the lack of any changes in duodenal sucrose activity or jejunal sucrose and maltase activity aligns with the normal developmental pattern, further investigation is required to determine ACR influence on enzyme activities during other critical periods that are well-established in normal developmental pattern. Moreover, understanding the mechanism of these changes is important. Intestinal lactase activity decreases with age due to changes in mRNA translation efficiency, increased protein degradation, the partial inactivation of the active site^32^. However, the specific mechanisms through which ACR influences these processes involved in the normal developmental pattern of enzyme activity are not yet known. The administration of ACR to pregnant dams may exert long-term effects that can be observed in lactating dams. It has been demonstrated that hormones such as thyroxine, thyroid-stimulating hormone, corticosterone, or adrenocorticotropin, which are present in the milk of dams starting from the third week of lactation, elicit responses in the offspring that result in changes in intestinal enzyme activities^33^.

Paneth cells located in crypts produce bactericidal secretions (microbicidal proteins including α-defensins, C-type lectins, lysozyme, and phospholipase A2)^34^, while specific endocrine Amine Precursor Uptake and Decarboxylation cells produce various hormones or biogenic amines, with endocrine or paracrine actions^35^. The secretory role of crypts could be disturbed after maternal ACR exposure, as evidenced by the time-dependent increase in number of total and inactive crypts in the PJ and ileum, which could be a compensation for the thinning and shallowing of crypts.

The same effect was observed in previously mentioned study on guinea pigs, where maternal ACR treatment significantly changed crypt morphology in duodenum and MJ. The number of total, dividing and inactive crypts increased, and crypts became shallower and thinner^19^.

Crypts also serve for the renewal and regeneration of the villi epithelium, through cell proliferation. Immature enterocytes rise from crypts and move along the lateral side of villi and undergo exfoliation on the villus` tip. These two processes should be in equilibrium to maintain proper villi length and shape. Even if the proliferation process is intense, shortening can be observed because of the massive exfoliation^36^. Villi morphology changes at weaning, when shortening is observed^37^. The time- and segment-dependent villi shortening in ACR-exposed rats, which occurred mainly in duodenum and ileum, could be a result of a disturbance in these two processes. Since our rats were at weaning age, prenatal ACR-exposure could have intensified the shortening process. To measure villous atrophy, the VH:CD ratio was used, which for humans ranges from 3:1 to 5:1^38^. In the current study, this parameter ranged from 2:1 to 9:1. It is not known if the range recommended for people can be extrapolated to rats. However, the changes in the VH:CD ratio in weaned rats exposed to ACR via their mothers were time- and segment-dependent.

ACR exposure could also evoke disturbances in absorption. An increase in the absorptive surface, in segment-dependent manner, was observed. Absorption depends on epithelium surface area and villi morphology, the architecture of which changes according to intestine segment, age or diet^39^. Villi are more numerous (and wider) in duodenum than in jejunum or ileum^40^, and this pattern was confirmed in current study. Maternal ACR exposure resulted in an increase in shorter and thinner villi. These changes were time- and segment-dependent, although not always statistically significant.

The study on prenatally ACR-exposed guinea pigs^19^ also showed an increase in villi number in the MJ and in damaged villi in the duodenum and jejunum, but contrary to the current study, a decrease in absorptive surface was also noted. Another study on postnatally ACR-exposed (25 mg/kg b.w./day) young rats showed shortening villi and epithelial damage in colon^41^. The authors suggested that degenerated villi, crypt injury and mucosal damage indicate impairment of absorption and secretion in colon^41^.

Since data on the effects of maternal ACR exposure on intestinal structure at weaning is very limited, the discussion is difficult or even impossible. Most studies focus on the developmental toxicological effects involving liver structure or function, blood cells or oxidative stress^27,42–44^. Limited studies performed on pregnant mice show changes in ovarian corpora lutea^45^ and placenta^46^. One study presents disturbances in skeletal system development in fetuses exposed to ACR (0.05, 1, 5 and 10 mg/kg b.w./day)^47^. Another study reports impairment of folliculogenesis in prenatally ACR-exposed females (3 mg/kg b.w./day)^48^. To our knowledge, there is only one study showing changes in intestinal structure of newborn offspring prenatally exposed to ACR at the same dose as that used in the current study^19^. Our previous study showed time-dependent effects of prenatal ACR exposure on oxidative stress, liver structure and function, and blood morphology in the same weaned rats^27^. There are numerous studies involving postnatal ACR administration. While some of the results are contradictory, they all show that ACR damages mitochondria and increases autophagy/apoptosis^49–51^, reactive oxygen species and pro-inflammatory factors^52,53^.

In the current study, apoptosis was investigated using the TUNEL method, for detection of apoptotic DNA fragmentation, allowing the in situ detection of AC in tissue samples^54^. The TUNEL assay showed a time- and segment-dependent increase in AC number. Numerous AC were found in the 10d ACR group, which was not accompanied by an increase in proliferation (more intensive after 5d ACR exposure). Moreover, all prenatally ACR-exposed rats had increased concentration of intestinal fabp2, an indicator of intestine damage^55,56^. Excessive proliferation (a sign of inflammation^40^), and intestinal wall leakage could lead to cancer^57^. Thus, the obtained results could suggest that maternal ACR-exposure may result in cancer later in life, according to the hypothesis of the prenatal origin of health and disease, being in line with fetal programming^58^. ACR is a well known carcinogen in rodents^59–61^,however, due to different metabolism and toxicokinetics in rodents and humans, ACR is not considered a human risk factor for cancer, which is supported by case–control and cohort studies^62,63^. Glycinamide, the main ACR metabolite in humans, is tumorigenic^64^. However, even though ACR intake in people is difficult to determine, its presence is detected in umbilical cord blood, and more attention is paid for prenatal ACR exposure leading to overweight in children^58^, that can be a risk factor of diabetes later in life^27^.

Based on previous results in guinea pigs, it was speculated that ACR given to pregnant dams disturbs intestinal regenerative properties, physiological function and stretching, due to a decrease in intestinal wall IC^19^. The current study also indicated a time-dependent change in collagen type. Collagen is an essential intestinal wall component and plays a beneficial role in the maintenance of a healthy gut and prevention of leaky gut^65^.

Taken together, the villi epithelium with microbiota, immune cells, goblet cells and tight junctions constitute the intestinal barrier^66^. Intestinal epithelial cells play a pivotal role in forming intestinal barrier involving immune and non-immune mechanisms that maintain intestinal homeostasis, equilibrium between noninvasive and invasive pathogens, food components and host humoral and cellular adaptive and innate immunity^67^. This barrier prevents leaky gut and microbial invasion, which can trigger carcinogenesis, according to a theory of driver-passenger pathogenesis of some intestinal cancers^68^. The increased gut permeability is caused by injury of the connection between enterocyte-like adherens and tight junctions^69^. Adherens junction proteins (E-cadherin) mediate cell–cell communication, while tight junction proteins (occludin and claudin) control passage of ions and small molecules through intestinal epithelium. Tight junctions are not permeable to high molecular weight molecules. This intercellular route is significantly restricted in ileum, while in duodenum and jejunum it is characterized by higher permeability, however the permeability in these sections is relative and depends on the osmotic and electrochemical gradient.

In the current study, the cadherin IR in untreated weaned rats was continuous in all intestinal segments, while it was discontinuous in all rats prenatally ACR-exposed. Moreover, the intensity of occludin IR was variable and time- and segment-dependent. Very similar effect was observed in newborn guinea pigs, namely interrupted and strongly punctate locally visible reaction in duodenum and MJ^19^.

In the current study immunodetection of leptin and ghrelin and their receptors was performed. The intensity of ghrelin IR increased in time-dependent manner in all segments of SI, except ileum (where it was decreased). Ghrelin receptor IR was decreased in general, however an increase was noted in duodenum in the 5d group. Leptin IR intensity changed in a time- and segment-dependent manner, while leptin receptor IR decreased in a time-dependent manner. It could indicate that maternal ACR exposure led to increased intestinal production of these two hormones, due to a decrease in their receptors.

Ghrelin is a protein hormone classified as an orexigen^70^. Ghrelin is synthetized in the stomach and throughout the whole SI and colon and released directly into the blood. The ghrelin receptor belongs to G-protein coupled receptor family and is found in many organs including the intestine^71^. For this reason it exerts local and systemic physiological processes^71^. It plays a key role in appetite stimulation and worsens glucose tolerance through reduction of insulin sensitivity in rodents and humans, thus increasing plasma glucose. Starvation results in an increase in plasma ghrelin, while food intake leads to a rapid decrease in plasma ghrelin in rodents and humans^72^. Ghrelin decreases and leptin increases in obese subjects, which is considered a physiological adaptation to obesity. Ghrelin induces opposite action to the leptin-induced decrease in food intake^73^. Leptin is also produced in the stomach and has various biological effects^74^. It initiates puberty, participates in immune and inflammatory responses, hematopoiesis, angiogenesis and bone formation. But, the most important role is a regulatory function in energy homeostasis and the control of body weight by the stimulation of metabolic rate and the suppression of food intake in rodents and humans^75^. It is proven that a decrease in plasma leptin in humans results in hyperphagia^73^.

Plasma leptin and ghrelin concentrations in the present study indicate that maternal ACR exposure decreases plasma ghrelin, irrespective of the duration of exposure, while an increase in plasma leptin was noted in the 10d group of rats. It should be emphasized that all rats were fasted, and plasma ghrelin still decreased. Moreover, plasma glucose concentration increased, with a tendency for decreased plasma insulin in rats prenatally ACR-exposed for 10 days^27^. If this is an adaptive mechanism to obesity, it should be investigated further, however, these rats have displayed a phenomenon called catch up growth, with their weight being comparable to control rats. A contrary effect was observed in other ACR-exposed rats^27^.

## Experimental section

### Ethics

The study was approved by the Local Ethics Committee for Animal Experiments (University of Life Sciences in Lublin, Poland, No. 88/2017). The methods were carried out in accordance with norms of the European Union law (Directive 2010/63/UE on the protection of animals used for scientific purposes, received in Poland by Legislative Decree 266/2015). The experiments were carried out at the Experimental Medicine Center of the Medical University of Lublin, Lublin, Poland in compliance with the ARRIVE guidelines.

### Reagents

All chemicals were purchased from Sigma-Aldrich (now owned by Merck KGaA, Darmstadt, Germany), unless otherwise stated.

### Animals

The study was carried out on offspring delivered by twenty-four adult female Wistar rats (initial body weight of approximately 220 g). The pregnant dams were kept individually in standard laboratory cages (55 cm × 33 cm × 20 cm) under standard laboratory conditions (a 12:12 day:night cycle, constant temperature of 22 ± 1 °C, room air renewals of 15–20 /h). Rats were fed standard laboratory rodents chow (Sniff Spezialdiäten GmbH, Soest, Germany) which met the nutritional requirements of AIN-93 diet^76^ with free access to drinking water. The same laboratory and feed conditions were maintained after delivery.

### Experimental groups

The pregnant dams were randomly divided into four groups: the control group (the 0d group, n = 6) and the three groups of pregnant dams exposed to ACR (3 mg/kg b.w./day, A8887, Sigma-Aldrich) for a period of either 15 days, (the 15d group, n = 6), 10 days (the 10d group, n = 6) or 5 days (the 5d group, n = 6), from the 6th, 11th or 16th day of pregnancy until parturition, respectively, as described in previous report^27^. The exposure to ACR was in accordance the following schedule (Fig. 1): Starting on the 6th day of pregnancy, rats from the 15d group, received ACR diluted in tap water by oral gavage, while the other rats were dosed with vehicle (tap water). The 10d group started to receive ACR on the 11th day of pregnancy, while the rats from 5 and 0d groups were still administered tap water. On the 16th day of pregnancy the 5d group started to receive ACR and the 0d group was still dosed with vehicle. Thus, ACR was given from the 6th, 11th or 16th day of pregnancy until parturition in the 5d, 10d and 15d groups, respectively. All dams were weighed daily and ACR solutions were adjusted every other day to account for changes in body weight. Parturition concluded ACR exposure, thus, neither mothers nor offspring were exposed to ACR during the rearing period. To normalize rearing conditions^77^, the culling procedure was performed on postnatal day 4, so that each mother had eight pups in total per litter, with four males and four females where possible^78^.

### Sampling

On postnatal day 21 (weaning), rats were fasted overnight, weighed, and one male and one female offspring from each dam (n = 12 offspring from each experimental group) with a body weight closest to litter average, were anesthetized with an intraperitoneal injection of ketamine/xylazine cocktail (100 mg + 10 mg per kg). Whole blood was collected by cardiac puncture into tubes with silica cloth activator (BD Vacutainer Systems, Plymouth, UK). Immediately after blood collection animals were sacrificed by cervical dislocation, SI segments were excised and rinsed thoroughly with saline. Sectioned parts of duodenum, proximal, middle and distal sections of jejunum and ileum were fixed in 10% buffered neutral formalin solution for histomorphometrical assessment and immunohistochemical evaluations. Adjacent to sectioned parts of duodenum and middle section of jejunum, fragments of intestine were opened, cleaned with saline and the mucosa was removed by gently scraping with a microscopic glass slide. Collected samples were immediately placed into tubes and stored in liquid nitrogen. The serum samples were prepared through centrifugation of coagulated blood at 1300× *g* for 10 min at 18 °C. Collected serum was aliquoted and stored at − 86 °C.

### Blood measurements

Blood serum concentrations of fatty acid binding protein 2 (fabp2), leptin and ghrelin were determined using commercial rat-specific enzyme-linked immunosorbent assay (ELISA) kits (#FY-ER6662, Feiyue Biotechnology, Wuhan, China; #QY-E11083 Qayee-bio, Shanghai, China; #CSB-E09816r, Cusabio, Wuhan, China, respectively). All assays were performed according to the manufacturers’ protocols with two technical replicates using a Benchmark Plus microplate spectrophotometer (Bio-Rad Laboratories, Inc., Hercules, CA, USA).

#### Determination of enzyme activity of intestinal brush border disaccharidases

The activities of lactase, maltase, and sucrase were determined according to the method described by Dahlquist^79^. Briefly, intestinal scrapings were first homogenized in chilled PBS, 100 μl of homogenate was incubated with 0.5 ml substrate (lactose, maltose, or sucrose solution) at 37 °C for 60 min, and the released glucose was quantified as described^79^. The amount of glucose released from the substrates was determined using the glucose assay and measured on microplate reader (Benchmark Plus, Bio-Rad Laboratories, Inc., Hercules, CA, USA). Activities were expressed as units (U) of μmol of disaccharide hydrolyzed in gram of mucosa per minute. The content of total protein in intestinal mucosa was determined using modified Lowry Protein Assay Kit (23240, Themofisher, Walthman, MA, USA) according to the manufacturer’s protocol.

### Histomorphometry

The SI samples after fixation in 10% formalin for 24 h were paraffin-embedded, cut in 4 µm sections, mounted on a microscope slide, stained with Goldner’s trichrome or Picrosirius red (PSR) and observed in standard bright illumination (Masson’s trichrome) or polarized light (PSR) using a light microscope (CX43, Olympus, Tokyo, Japan). All histological examinations were undertaken by a very experienced associate who was blinded to the study protocol. Microscopic observations of Goldner’s trichrome stained sections were used for the assessment of the histomorphometry of intestine samples. The thickness of the mucosa, submucosa and both myenterons, the number of villi and crypts per mm of mucosa and the number of enterocytes and goblet cells were determined. The length, width and surface of intestinal villi and the length and width of the crypts were measured^80^. Special attention was paid to the correct orientation and only sections containing longitudinally cut villus and crypts were accepted for analyses. Quantitative villus height/crypt depth ratio and mucosal surface absorptive area, expressed as mucosal-to-serosal amplification ratio^81^, were calculated for individual villi-crypt pairs. For each parameter, 10 replicate measurements were taken per animal. The analyses were performed using ImageJ software^82^. PSR staining was used distinguish between type I and type III collagen in examined SI samples, where type I (mature) collagen fibres appear orange or red, and type III (immature) fibres appear green in polarized light^83,84^.

### Immunohistochemical analyses

The immunohistochemical analysis was performed with rabbit anti-E-cadherin (#AF0131, Affinity Biosciences, Jiangsu, China), anti-occludin (#DF7504, Affinity Biosciences), anty-leptin (#DF8583, Affinity Biosciences), anti-leptin receptor (#DF7139, Affinity Biosciences), anti-ghrelin (#DF6389, Affinity Biosciences), anti-ghrelin receptor (#DF2794, Affinity Biosciences) as primary antibodies diluted (1:100) in Diamond antibody diluent (Cell Marque Corp., Rocklin, CA, USA). The sections were then processed with ready-to-use two-step detection system PolyHRP-Goat Anti-Mouse/Rabbit IgG (#DPVB110HRP, BrightVision, Immunologic WellMed B.V., Duiven, Netherlands), developed in DAB (#D5905, Sigma-Aldrich) and counterstained which Mayer’s hematoxylin (#MHS32, Sigma-Aldrich). Prior the quantitative measurements of the intensity of IR images acquired using a CX43 and d BX63 light microscopes (Olympus, Tokyo, Japan) were converted into 8-bit grey-scale images. The calculation of the optical density (OD) was performed according the ImageJ protocol, using Kodak 3 step tablet for calibration and ImageJ build-in Rodbard function to convert mean 8-bit pixel value to calibrated OD score^82^. The measurements were carried out separately for villus and crypts in five randomly selected areas of the positive signal using ImageJ software^82^ by an associate who was who was not aware of the treatment.

Immunohistochemical analysis of Ki-67 as a marker of cell proliferation was performed using rabbit anty-Ki-67 (ab15580, AbCam, Cambridge, UK) in the same manner as described above. The number of positively stained nuclei for Ki-67 per area of the gland surface was counted from at 5 areas per rat.

### TUNEL reaction

To detect apoptotic cells TUNEL (terminal deoxynucleotidyl transferase-mediated dUTP nick-end labeling) staining was carried out on paraffin sections using ApopTag Peroxidase In Situ Apoptosis Detection Kit (Chemicon, Merck KGaA, Darmstadt, Germany) according to the manufacturer's protocol, developed in DAB and counterstained which Mayer’s hematoxylin as described previously^85^. For negative control, terminal deoxynucleotidyl transferase was omitted, for positive control, sections of piglet’s ovary showing apoptotic oocytes were selected. The slides were examined and photographed using an a CX43 light microscope (Olympus, Tokyo, Japan). TUNEL-positive cells were counted in ten non-overlapping microscopic fields. Only dark brown stained cells were interpreted as TUNEL-positive apoptotic cells. The counting of apoptotic cells was performed by a blinded associate and normalized to the examined area of the intestine cross-section.

### Statistical analysis

All statistical procedures were conducted using the GLM MIXED procedure of SAS (SAS Institute. Inc., Cary, NC, USA) with prenatal ACR exposure as fixed effect and mother as random effect to correct for differences between the litters. The linear mixed-effects model allowed for considering an individual rat (n = 12 per group) as the experimental unit of analysis and treated the litter as a random variable in the ANOVA^86,87^. When significantly different, a post hoc Tukey’s HSD adjustment was used to compare the means. A *p* value of < 0.05 was considered statistically significant for all comparisons. Data are expressed as the least squares means (lsmeans) and standard error of means (Table 1 and supplementary Table S1) or standard deviation (figures). In figures, each datapoint represent the lsmeans value of the calculated parameters for each rat.

## Conclusions

In summary, this is the first study showing the influence prenatal ACR exposure on postnatal development of the SI and disturbed adherent and tight junction of intestine in time- and segment-dependent manner. ACR also was responsible for the changes of the activity of intestinal brush border disaccharidases in time- and segment-dependent-manner.

## Supplementary Information

Supplementary Table S1.

## Data Availability

The datasets used and analyzed during the current study are available from the corresponding author on reasonable request.

## Abbreviations

AC: Apoptotic cell
ACR: Acrylamide
ANOVA: One‐way analysis of variance
BER: Basic electrical rhythm
b.w.: Body weight
DAB: 3, 3'-Diaminobenzidine
DJ: Distal jejunum
fabp2: Fatty acid binding protein 2
GIT: Gastrointestinal tract
IC: Immature collagen
IR: Immunoreaction
Lsmeans: Least squares means
MJ: Middle jejunum
ML: Muscular lamina
PJ: Proximal jejunum
PSR: Picrosirius red
SEM: Standard error of means
SI: Small intestine
TUNEL: Terminal deoxynucleotidyl transferase-mediated dUTP nick-end labeling
VH:CD ratio: Villus height/crypt depth ratio
