# Supplementary material for: Maternal acrylamide exposure changes intestinal epithelium, immunolocalization of leptin and ghrelin and their receptors, and gut barrier in weaned offspring
Source: Sci Rep. 2023 Jun 24;13:10286. doi: 10.1038/s41598-023-37590-3 (PMC10290718; doi:10.1038/s41598-023-37590-3)
Supplement: Supplementary file 1 — Supplementary Table S1. [file 41598_2023_37590_MOESM1_ESM.docx]

Table S1. Basal morphological parameters of small intestine sections of weaned Wistar rats not prenatally exposed to acrylamide (0d) or exposed to acrylamide for 5, 10 or 15 days of prenatal life up to parturition.

|  | **0d** | **5d** | **10d** | **15d** | **p-value** |
| --- | --- | --- | --- | --- | --- |
| *Duodenum* | | | | | |
| Mucosa thickness, μm | 520 ± 21 | 558±22 | 543 ± 33 | 507 ± 44 | 0.739 |
| Submucosa thickness, μm | 22.62 ± 2.52 ^a^ | 24.08 ± 1.51 ^ab^ | 38.96 ± 6.86 ^b^ | 18.06 ± 1.26 ^a^ | 0.005 |
| Longitudinal lamina thickness, μm | 19.27 ± 2.13 | 19.26 ± 0.59 | 19.83 ± 1.53 | 18.79 ± 1.51 | 0.972 |
| Transversal lamina thickness, μm | 33.11 ± 2.79 | 31.54 ± 1.99 | 36.74 ± 2.82 | 32.74 ± 2.26 | 0.503 |
| Total number of villi, mm^-1^ | 6.92 ± 0.19 | 7.67 ± 0.38 | 7.50 ± 0.29 | 7.83 ± 0.32 | 0.191 |
| Villus length, µm | 647 ± 4 ^c^ | 479 ± 4 ^a^ | 555 ± 7 ^b^ | 673 ± 12 ^c^ | <0.001 |
| Villus thickness, µm | 78.68 ± 1.67 ^c^ | 35.20 ± 0.97 ^a^ | 69.75 ± 1.96 ^b^ | 79.66 ± 1.08 ^c^ | <0.001 |
| Villus volume, µm^3^ | 159.9 ± 3.3 ^c^ | 53.0 ±1.5 ^a^ | 121.4 ±3.2 ^b^ | 168.5 ± 2.5 ^c^ | < 0.001 |
| Goblet cells number, (100 µm of villus)^-1^ | 4.58 ± 0.29 | 4.67 ± 0.26 | 4.92 ± 0.40 | 4.42 ± 0.44 | 0.782 |
| Enterocyte number, (100 µm of villus)^-1^ | 18.72 ± 0.33 ^ab^ | 17.12 ± 0.43 ^a^ | 19.06 ± 0.57 ^b^ | 18.56 ± 0.52 ^ab^ | 0.042 |
| Villus epithelium thickness, µm | 25.34 ± 0.67 | 22.83 ± 0.51 | 23.67 ± 0.87 | 24.22 ± 0.57 | 0.092 |
| Total crypts number, mm^-1^ | 2.85 ± 0.15 | 3.72 ± 0.11 | 3.09 ± 0.11 | 2.74 ± 0.13 | 0.245 |
| Active crypts number, mm^-1^ | 0.336 ± 0.044 | 0.297 ± 0.52 | 0.225 ± 0.072 | 0.393 ± 0.060 | 0.333 |
| Inactive crypts number, mm^-1^ | 2.52 ± 0.15 | 2.40 ± 0.12 | 2.84 ± 0.14 | 2.33 ±0.14 | 0.106 |
| Crypt depth, μm | 134.7 ± 1.0 ^c^ | 56.26 ± 3.56 ^a^ | 88.96 ± 1.62 ^b^ | 140.99 ± 2.48 ^c^ | <0.001 |
| Crypt thickness, µm | 45.27 ± 0.72 ^d^ | 26.60 ± 0.54 ^b^ | 20.69 ± 0.21 ^a^ | 39.76 ± 0.30 ^c^ | <0.001 |
| Villus:crypt ratio, -- | 4.81 ± 0.04 ^a^ | 9.05 ± 0.72 ^b^ | 6.26 ± 0.16 ^a^ | 4.80 ± 0.16 ^a^ | <0.001 |
| Mucosal surface absorptive area, -- | 13.92 ± 0.17 ^a^ | 18.58 ± 0.25 ^c^ | 19.59 ± 0.51 ^c^ | 15.72 ± 0.27 ^b^ | < 0.001 |
| *Proximal jejunum* | | | | | |
| Mucosa thickness, μm | 419.4 ± 30 | 345.0 ± 22 | 358.7 ± 21 | 347.1 ± 20 | 0.119 |
| Submucosa thickness, μm | 17.91 ± 1.91 | 16.60 ± 1.26 | 19.47 ± 1.74 | 18.23 ± 1.10 | 0.630 |
| Longitudinal lamina thickness, μm | 17.32 ± 1.65 | 16.99 ± 1.32 | 18.99 ± 1.05 | 19.23 ± 1.39 | 0.567 |
| Transversal lamina thickness, μm | 27.24 ± 2.98 | 23.65 ± 3.84 | 21.54 ± 1.79 | 25.08 ± 2.51 | 0.567 |
| Total number of villi, mm^-1^ | 7.92 ± 0.53 ^a^ | 9.75 ± 0.66 ^ab^ | 10.0 ± 0.69 ^ab^ | 10.83 ± 0.52 ^b^ | 0.040 |
| Villus length, µm | 358.25 ± 14 | 383.11 ± 18 | 389.37 ± 23 | 360.91 ± 21 | 0.580 |
| Villus thickness, µm | 97.14 ± 6.59 | 93.32 ± 3.23 | 88.01 ± 5.47 | 84.15 ± 7.74 | 0.482 |
| Villus volume, µm^3^ | 109.4 ± 8.6 | 113.8 ± 6.3 | 107.0 ±8.7 | 92.5 ±6.9 | 0.326 |
| Goblet cells number, (100 µm of villus)^-1^ | 7.42 ± 0.70 | 8.33 ± 0.88 | 9.58 ±0.99 | 8.25 ± 1.06 | 0.437 |
| Enterocyte number, (100 µm of villus)^-1^ | 16.13 ± 0.37 ^a^ | 17.89 ± 0.40 ^b^ | 18.74 ± 0.53 ^b^ | 18.55 ± 0.38 ^b^ | 0.001 |
| Villus epithelium thickness, µm | 18.92 ± 0.43 | 19.57 ± 0.42 | 18.83 ± 0.75 | 19.31 ± 0.64 | 0.790 |
| Total crypts number, mm^-1^ | 2.64 ± 0.15 ^a^ | 3.28 ± 0.13 ^b^ | 3.29 ± 0.13 ^b^ | 2.95 ± 0.16 ^ab^ | 0.021 |
| Active crypts number, mm^-1^ | 0.341 ±0.063 | 0.315 ± 0.061 | 0.285 ± 0.052 | 0.248 ± 0.081 | 0.780 |
| Inactive crypts number, mm^-1^ | 2.30 ± 0.18 ^a^ | 3.00 ± 0.11 ^b^ | 3.01 ± 0.14 ^b^ | 2.67 ± 0.13 ^ab^ | 0.008 |
| Crypt depth, μm | 111.7 ± 6.4 ^c^ | 94.4 ± 4.4 ^bc^ | 86.8 ± 4.3 ^ab^ | 69.1 ± 3.6 ^a^ | <0.001 |
| Crypt thickness, µm | 34.80 ± 1.18 ^b^ | 25.99 ± 0.93 ^a^ | 26.36 ± 0.60 ^a^ | 28.34 ± 1.24 ^a^ | <0.001 |
| Villus:crypt ratio, -- | 3.33 ± 0.24 ^a^ | 4.15 ± 0.26 ^ab^ | 4.51 ± 0.20 ^ab^ | 5.51 ± 0.62 ^b^ | 0.005 |
| Mucosal surface absorptive area, -- | 8.76 ± 0.52 | 10.74 ± 0.54 | 11.35 ± 0.71 | 10.74 ± 98 | 0.088 |
| *Middle jejunum* | | | | | |
| Mucosa thickness, μm | 314.13 ± 24 | 338.35 ± 15 | 306.45 ± 19 | 286.23 ± 19 | 0.399 |
| Submucosa thickness, μm | 15.77 ± 0.93 ^b^ | 9.39 ± 0.59 ^a^ | 13.37 ± 1.33 ^ab^ | 15.03 ± 1.35 ^b^ | 0.049 |
| Longitudinal lamina thickness, μm | 16.09 ± 1.33 | 13.63 ± 1.15 | 15.35 ± 1.02 | 16.42 ± 1.31 | 0.391 |
| Transversal lamina thickness, μm | 27.62 ± 3.76 | 22.68 ± 2.07 | 21.21 ± 1.87 | 22.10 ± 1.63 | 0.284 |
| Total number of villi, mm^-1^ | 8.75 ± 0.71 | 7.92 ± 0.48 | 7.92 ± 0.42 | 8.67 ± 0.59 | 0.615 |
| Villus length, µm | 345.8 ± 17 ^ab^ | 286.4 ± 12 ^a^ | 315.5 ± 10 ^ab^ | 362.5 ± 25 ^b^ | 0.025 |
| Villus thickness, µm | 102.75 ± 5 | 93.17 ± 4 | 90.46 ± 3 | 93.35 ± 6 | 0.429 |
| Villus volume, µm^3^ | 112.6 ± 7.7 | 84.3 ± 5.7 | 90.2 ±5.1 | 104.7 ±8.5 | 0.069 |
| Goblet cells number, (100 µm of villus)^-1^ | 11.25 ± 0.66 | 8.17 ± 051 | 8.67 ± 0.75 | 9.17 ± 1.29 | 0.095 |
| Enterocyte number, (100 µm of villus)^-1^ | 18.00 ± 0.32 ^a^ | 19.93 ± 0.40 ^b^ | 19.19 ± 0.40 ^a^ | 18.58 ± 0.24 ^a^ | 0.006 |
| Villus epithelium thickness, µm | 19.70 ± 0.58 | 19.22 ± 0.42 | 18.30 ± 0.67 | 18.96 ± 0.64 | 0.429 |
| Total crypts number, mm^-1^ | 2.72 ± 0.16 | 2.86 ± 0.14 | 2.85 ± 0.10 | 3.01 ± 0.11 | 0.574 |
| Active crypts number, mm^-1^ | 0.215 ± 0.076 | 0.436 ± 0.102 | 0.411 ± 0.066 | 0.487 ± 0.084 | 0.137 |
| Inactive crypts number, mm^-1^ | 2.51 ± 0.21 | 2.42 ± 0.13 | 2.44 ± 0.15 | 2.51 ± 0.16 | 0.969 |
| Crypt depth, μm | 113.94 ± 6.84 ^b^ | 88.75 ± 7.47 ^a^ | 86.28 ± 4.51 ^a^ | 95.25 ± 3.39 ^ab^ | 0.013 |
| Crypt thickness, µm | 35.35 ± 1.35 ^b^ | 27.67 ± 1.24 ^a^ | 27.71 ± 1.02 ^a^ | 27.69 ± 0.47 ^a^ | <0.001 |
| Villus:crypt ratio, -- | 3.11 ±0.19 | 3.43 ± 0.25 | 3.71 ± 0.14 | 3.83 ± 0.29 | 0.147 |
| Mucosal surface absorptive area, -- | 8.14 ± 0.40 ^ab^ | 7.97 ± 0.37 ^a^ | 8.73 ± 0.29 ^ab^ | 10.11 ± 0.98 ^b^ | 0.033 |
| *Distal jejunum* | | | | | |
| Mucosa thickness, μm | 310.7 ± 17 ^b^ | 308.7 ± 9 ^ab^ | 253.8 ± 9 ^a^ | 302.5 ± 11 ^ab^ | 0.031 |
| Submucosa thickness, μm | 15.35 ± 0.93 | 14.72 ±1.15 | 12.34 ± 0.59 | 14.06 ± 0.67 | 0.114 |
| Longitudinal lamina thickness, μm | 15.84 ± 0.93 ^ab^ | 18.01 ± 1.34 ^b^ | 12.99 ± 0.67 ^a^ | 14.07 ± 0.58 ^ab^ | 0.016 |
| Transversal lamina thickness, μm | 26.49 ± 1.13 | 24.75 ± 1.37 | 22.35 ± 1.30 | 23.92 ± 1.33 | 0.246 |
| Total number of villi, mm^-1^ | 6.58 ± 0.29 | 7.34 ± 0.51 | 7.12 ± 0.47 | 7.12 ± 0.27 | 0.587 |
| Villus length, µm | 233.0 ± 12 | 233.2 ± 11 | 207.7 ± 5 | 238.8 ± 10 | 0.276 |
| Villus thickness, µm | 99.99 ± 4.18 | 93.37 ± 4.27 | 85.93 ± 4.26 | 96.03 ± 3.13 | 0.135 |
| Villus volume, µm^3^ | 73.9 ± 5.8 | 69.7 ±5.4 | 57.0 ±3.9 | 72.8 ± 5.1 | 0.176 |
| Goblet cells number, (100 µm of villus)^-1^ | 8.42 ± 0.58 | 8.67 ± 0.54 | 10.00 ± 0.46 | 8.58 ± 0.57 | 0.307 |
| Enterocyte number, (100 µm of villus)^-1^ | 18.51 ± 0.62 ^a^ | 19.91 ± 0.34 ^a^ | 21.89 ± 0.35 ^b^ | 20.22 ± 0.23 ^a^ | <0.001 |
| Villus epithelium thickness, µm | 17.49 ± 0.43 | 16.65 ± 0.57 | 17.59 ± 0.48 | 17.31 ± 0.49 | 0.600 |
| Total crypts number, mm^-1^ | 2.58 ± 0.11 ^a^ | 3.05 ±0.14 ^b^ | 2.82 ± 0.12 ^a^ | 2.99 ± 0.06 ^a^ | 0.036 |
| Active crypts number, mm^-1^ | 0.156 ± 0.052 | 0.352 ± 0.091 | 0.254 ±0.072 | 0.278 ± 0.057 | 0.285 |
| Inactive crypts number, mm^-1^ | 2.42 ± 0.11 | 2.70 ± 0.18 | 2.57 ± 0.13 | 2.72 ±0.10 | 0.395 |
| Crypt depth, μm | 110.96 ± 4. 94 ^c^ | 99.80 ± 2.20 ^bc^ | 82.41 ± 2.23 ^a^ | 89.34 ± 3.83 ^ab^ | <0.001 |
| Crypt thickness, µm | 33.17 ± 0.64 | 30.84 ± 0.50 | 31.55 ± 1.21 | 31.09 ± 0.96 | 0.397 |
| Villus:crypt ratio, -- | 2.12 ± 0.10 ^a^ | 2.36 ± 0.16 ^ab^ | 2.55 ± 0.11 ^ab^ | 2.72 ± 0.16 ^b^ | 0.038 |
| Mucosal surface absorptive area, -- | 5.77 ± 0.25 | 6.14 ± 0.27 | 5.73 ± 0.15 | 6.19 ± 0.20 | 0.401 |
| *Ileum* | | | | | |
| Mucosa thickness, μm | 232.17 ± 11 | 217.72 ± 21 | 212.75 ± 11 | 195.30 ± 15 | 0.399 |
| Submucosa thickness, μm | 10.22 ± 0.63 | 11.16 ± 0.81 | 11.33 ± 0.94 | 16.69 ± 3.35 | 0.079 |
| Longitudinal lamina thickness, μm | 16.86 ± 1.77 | 17.72 ± 2.12 | 17.18 ± 1.64 | 15.57 ± 0.93 | 0.900 |
| Transversal lamina thickness, μm | 24.84 ± 2.70 | 25.1 ± 3.37 | 27.25 ± 2.19 | 25.32 ± 2.11 | 0.935 |
| Total number of villi, mm^-1^ | 4.92 ± 0.40 ^a^ | 7.25 ± 0.33 ^b^ | 6.50 ± 0.48 ^ab^ | 6.42 ± 0.48 ^ab^ | 0.008 |
| Villus length, µm | 188.5 ± 9 | 170.6 ± 9 | 192.2 ± 7 | 172.9 ± 8 | 0.430 |
| Villus thickness, µm | 77.98 ± 5.11 | 72.34 ± 4.14 | 84.82 ± 3.82 | 72.36 ± 5.46 | 0.308 |
| Villus volume, µm^3^ | 45.6 ± 3.1 | 40.0 ±4.1 | 48.6 ±2.7 | 40.8 ± 4.2 | 0.316 |
| Goblet cells number, (100 µm of villus)^-1^ | 6.25 ± 0.49 | 4.83 ± 0.32 | 5.25 ± 0.45 | 4.67 ± 0.31 | 0.114 |
| Enterocyte number, (100 µm of villus)^-1^ | 20.17 ± 0.27 | 20.58 ± 0.39 | 21.07 ± 0.36 | 20.52 ± 0.48 | 0.553 |
| Villus epithelium thickness, µm | 16.81 ± 0.38 ^ab^ | 17.34 ± 0.66 ^b^ | 15.19 ± 0.35 ^a^ | 15.71 ± 0.30 ^ab^ | 0.030 |
| Total crypts number, mm^-1^ | 2.24 ± 0.15 | 2.80 ± 0.14 | 2.71 ± 0.15 | 2.60 ± 0.20 | 0.122 |
| Active crypts number, mm^-1^ | 0.197 ± 0.054 | 0.274 ± 0.070 | 0.344 ± 0.091 | 0.264 ± 0.051 | 0.644 |
| Inactive crypts number, mm^-1^ | 2.04 ± 0.13 | 2.53 ± 0.15 | 2.36 ± 0.18 | 2.33 ± 0.19 | 0.285 |
| Crypt depth, μm | 81.22 ± 4.36 | 89.01 ± 6.89 | 94.18 ± 3.85 | 81.86 ± 5.37 | 0.736 |
| Crypt thickness, µm | 38.28 ± 1.22 ^b^ | 30.60 ± 1.38 ^a^ | 33.14 ± 1.79 ^ab^ | 31.68 ± 1.43 ^a^ | 0.015 |
| Villus:crypt ratio, -- | 2.41 ± 0.19 | 2.03 ± 0.19 | 2.20 ± 0.14 | 2.22 ± 0.20 | 0.617 |
| Mucosal surface absorptive area, -- | 4.95 ± 0.24 | 5.23 ±0.30 | 5.04 ± 0.22 | 5.18 ± 0.18 | 0.877 |

Data are presented as lsmeans ± SEM (standard error of mean). Rows with different superscripts (^a, b, c^) are significantly different at *p*-value < 0.05 (GLM MIXED procedure with an individual rat as the experimental unit, n = 12 per group, and a post hoc Tukey’s HSD adjustment).
